# Effects of Frugivore Preferences and Habitat Heterogeneity on Seed Rain: A Multi-Scale Analysis

**DOI:** 10.1371/journal.pone.0033246

**Published:** 2012-03-16

**Authors:** Javier Rodríguez-Pérez, Asier R. Larrinaga, Luis Santamaría

**Affiliations:** 1 Institut Mediterrani d'Estudis Avançats, IMEDEA, Mallorca, Balearic Islands, Spain; 2 UFZ Helmholtz Centre for Environmental Research, Umweltforschungszentrum, Department of Ecological Modeling, Leipzig, Germany; Michigan State University, United States of America

## Abstract

Seed rain mediated by frugivores is influenced by (1) the seed-deposition distances following fruit ingestion, (2) the disperser activity, as determined by its behaviour and habitat preferences, and (3) the structure of the habitat within the landscape. Here, we evaluated such components using the fleshy-fruited shrub *Ephedra fragilis* and the frugivorous Balearic lizard *Podarcis lilfordi*. We estimated seed-deposition patterns based on the displacements and habitat preferences of lizards, derived from visual surveys and telemetry data. The influence of variables potentially determining lizard habitat preference (i.e., height, slope, four measures of habitat abundance and four measures of habitat fragmentation) was evaluated at three spatial scales: ‘home-range’ (c. 2.5–10*10^3^ m^2^; telemetry data), ‘within home-range’ (c. 100 m^2^; telemetry data) and ‘microhabitat’ (<100 m^2^; visual survey). Cumulative lizard displacement (from each telemetric location to the initial capture point) saturated before the peak of seed defecation (seed-retention time), indicating that lizard home-range size and habitat preferences were the main determinants of the spread and shape of seed shadows. Shrub cover was positively correlated with habitat preference at the three scales of analysis, whereas slope was negatively correlated at the home-range scale. Model scenarios indicated that spatially-aggregated seed rain emerged when we incorporated the joint effect of habitat preference at the two largest (home-range and within home-range) scales. We conclude that, in order to predict seed rain in animal dispersed plants, it is important to consider the multi-scale effects of habitat preference by frugivores.

## Introduction

Seed dispersal is crucial for the plant regeneration process and sets the template on which the structure and dynamics of plant populations operate [1], [2]. The dispersal capacity of plant species results from the combination of three factors: the intrinsic characteristics (traits) of the dispersal unit (hereafter referred to as ‘seeds’), the contribution of the various dispersal vectors and the spatial structure of the habitat [3].

In the case of animal-dispersed plants, dispersal capacity will depend on the interplay between gut-passage time of seeds and the disperser's movement pattern [2], [4]–[6]. At one extreme, when the movement of seed dispersers is predominantly directional (i.e., animals with large home-ranges or moving between fragmented areas) [7], seed shadows will depend on both the gut-passage time and the movement characteristics (speed and direction) of the disperser [8]. At the other extreme, when frugivores rapidly cover and revisit a restricted area (i.e., territorial animals with small home-ranges and fast displacements), their displacement from the seed-ingestion point saturates before seed defecation; hence, seed shadows will depend on the size and shape of the disperser's home-range and its habitat preferences [9]. If we want to disentangle which one of the two components (the disperser's physiology and/or behaviour) is more important for the seed dispersal process, we have to weight the importance of the temporal (seed retention time) and spatial factors (maximum frugivore displacement) at which seed dispersal operates.

The spatial distribution of animals is also affected by the structure of the habitat mosaic within the landscape [10]. Animals explore the space in a non-random fashion, which mainly depends on their displacement capacity (step distance), their requirements (food, shelter, breeding sites) and their previous knowledge of the area. Habitat selection may be thus envisaged as a hierarchical spatial process, encompassing a range of decisions that span from the characteristics (size, shape) of a given home-range to the choice of specific dietary units [11]–[12]. Such hierarchical selection processes have direct consequences for animal-dispersed plants, whose seeds are often dispersed to microhabitats or locations highly preferred by frugivores [13]–[14]. Seed shadows may therefore be moulded by two components that are strongly interlinked: the scale of habitat heterogeneity, which sets the template for resource variability, and the habitat preferences of frugivores, which translate habitat variability into a heterogeneous use of the space they inhabit [10], [15]. Still, an inspection of the literature shows that seed dispersal was traditionally viewed as a process affecting plant populations that is independent of the specific characteristics of animal activity and behaviour - and, though recent works have focused in incorporating these components, they rarely do so from a spatially-explicit perspective [14], [16]–[20]. As a consequence, we still lack data on the spatial scales at which animal-mediated dispersal processes operate in natural ecosystems and their implications for plant populations [6].

We present here a study of the plant-disperser interaction between the fleshy-fruited shrub *Ephedra fragilis* Desf. (Ephedraceae) and the frugivorous lizard *Podarcis lilfordi* Günter (Lacertidae), carried out during a masting year of the plant partner. Our work focuses on identifying both the main determinants of *Ephedra* seed dispersal and the spatial scales at which these determinants could influence seed shadows. For this purpose, we address the following questions:

1. Is seed-dispersal distance primarily determined by lizard displacement (i.e., speed and distance from the source point, combined with seed gut-passage time) or by its habitat preferences (home-range size and shape, habitat preferences)?
2. What are the habitat preferences of lizards and at which spatial scales do they operate? We considered three spatial scales: home-range (10^3^–10^4^ m^2^), within home-range (c. 100 m^2^) and microhabitat (<100 m^2^).
3. What are the potential effects of lizard habitat preferences on the seed rain? Do habitat preferences operating at different spatial scales show comparable effects on the seed rain?

We addressed these questions using scenarios based on lizard habitat preferences, to simulate the resulting seed rain. It is important to note that the purpose of this habitat model is purely heuristic, instead of predictive - i.e., it is exclusively aimed at exploring the consequences of disperser behaviour patterns for seed rain, not at predicting their specific shape.

## Materials and Methods

### Study system

*Ephedra fragilis* (*Ephedra* hereafter) is a dioecious evergreen shrub (up to 4 m in height) inhabiting the (semi)arid sclerophyllous shrublands of the Western Mediterranean basin and the Macaronesian region. In masting years, female plants can produce more than 10,000 fruits, which are available from June to September (unpubl. data). Arillated fruits (fruits hereafter) have red or yellow arils and bear only one seed (length: 4.87±0.11 mm; width: 2.10±0.06 mm; weight: 14.31±0.64 mg; n = 233). They are consumed by birds [21] and, in the Balearic Islands, by lizards [22]. Owing to the low number of resident passerines during summer, Balearic lizards represent the almost-exclusive disperser of Ephedra seeds in some islets of the Balearic Islands (such as Dragonera Islet; unpubl. data). Ephedra plants reproduce in episodic events of massive fruit production (i.e., masting years; pers. obs.) and recruits are extremely rare in natural populations (pers. obs.), which suggests that environmental conditions strongly limit its regeneration (e.g., [23] for another Ephedra species).

The Balearic lizard, *Podarcis lilfordi*, is a small diurnal lizard (snout-vent length: ♂ 6.67 cm, ♀ 5.8 cm; weight: ♂ 9.7 g and ♀ 5.8 g; *n* = 77) endemic to the Western Balearic Islands (Mallorca and Menorca; Western Mediterranean) and closely related to the Eastern Balearic lizard *Podarcis pityusensis* (endemic to Eivissa and Formentera). Both species are important pollinators and seed dispersers of the native flora [22].

### Study site – description of habitat structure

Field work took place at Dragonera, a small (c. 300 ha) islet situated 1,300 m offshore Mallorca Island (Fig. S1), during an *Ephedra* masting year (i.e., with high fruit availability). In masting years, *Ephedra* fruits are a predominant component of the diet of Balearic lizards at this population (e.g., during a pilot study, c. 80% of lizard defecations had *Ephedra* pulp and seeds, and this proportion was comparable for both males and females: χ^2^_1_ = 1.02, *p* = 0.313, *n* = 80). Our survey took place from 20^th^ to 25^th^ of July 2004, coinciding with the peak of *Ephedra* fruit production and with the period of maximum activity of Balearic lizards (June–August; pers. obs.). The study site was located in a stony, steep slope facing southeast and located between the shoreline and 80 m a.s.l. The landscape is dominated by small soil pockets and rock outcrops that provide abundant refuges for lizards, interspersed with patches of open sclerophyllous shrubland dominated by *Ephedra*, *Pistacia lentiscus* and *Phillyrea angustifolia*. *P. lilfordi* is the only frugivorous reptile inhabiting Dragonera.

The spatial analysis of habitat characteristics and lizard preferences was based on an observation unit (‘grain’) of 12×12 m. The size of these units was chosen to match the spatial resolution of our lizard telemetry locations, which showed a median error of 11.6±0.3 m (Fig. S2; see below). Hence, the study area was subdivided in a lattice of 110 grid-cells of 144 m^2^ (Fig. S1) and environmental variables were extracted, for each grid-cell, from a digital elevation model (DEM) and a habitat map. We obtained the DEM from 1∶1000 cartography, while the habitat map was derived from the supervised classification of an aerial ortho-photograph, with four habitat types (rock, bare soil, sclerophillous shrub and *Ephedra* shrub). For each grid-cell, we estimated two topographic variables (height and slope, derived from the DEM), two habitat variables (the proportions of rock and shrub; Fig. S1) and four variables describing landscape fragmentation (Text S1): (a) number of shrub patches, (b) mean size of shrub patches, (c) mean shape of shrub patches (patch perimeter divided by the perimeter of a circle of identical area) and (d) mean distance to the closest shrub patch. We estimated these fragmentation measures using the software FRAGSTATS (McGarigal 2002).

### Retention time and germination of seeds

During the morning of July 20^th^ of 2004, 15 males and 13 females of Balearic lizard were captured using pit-fall traps baited with tomato. Captures took place approx. 1,500 m away from the study site, at an area free of *Ephedra* plants, in order to avoid (a) influencing the abundance and behaviour of the lizards of our study site, and (b) capturing lizards with recently-ingested *Ephedra* seeds in their guts. Lizards were kept in individual *terraria* with the bottom covered with artificial grass and a piece of brick as a refuge, placed in a nearby, quiet and shaded site. Within that site, the spatial arrangement of the individual *terraria* was randomised.

During the morning of the second day, all lizards were force-fed with *Ephedra* fruits from our study site. Force-feeding was considered necessary to achieve comparable, controlled and synchronic feeding events, which is a pre-requisite to accurately estimate seed retention time [9]. Fruits were collected from 14 plant individuals. Each individual was randomly assigned to one male and one female lizard (except for one plant, which was assigned to two males). A group of non-ingested fruits was also set aside from each plant individual, to be used as controls for germination experiments (see below). Each lizard ingested, on average, 3.89±0.13 (mean ± SE) fruits, although the specific number fed to each individual varied with its size (i.e., more fruits were fed to larger lizards) and willingness to be fed (to minimize force-feeding stress and keep feeding times <5 min). Lizards were provided with food (tomato) and water *ad libitum* for the rest of the experiment, and regularly inspected (in the morning, 8–9 a.m., early afternoon, 3–4 p.m., and late afternoon, 8–9 p.m.) to collect newly-produced faeces and separate all defecated seeds. We did not check the *terraria* at night because lizards were inactive and did not produce any faeces (unpubl. data, during a pilot experiment). Defecated seeds were gently cleaned, dried with blotting paper, weighed (±0.1 mg) and stored individually at dry place at room temperature (20–25°C).

On December 7^th^ of 2004, defecated seeds were sown in an experimental garden (mainland Mallorca, 22 km away from Dragonera), together with non-ingested, depulped control seeds from the same *Ephedra* individuals (*n* = 10 seeds per plant). Seeds were sowed at approx. 5 mm depth in germination trays of 60 (4×4 cm) pots (one seed per pot), filled with horticultural mixture and watered automatically 2–3 times per day. Seed germination was monitored at weekly intervals during one year. Seeds that failed to germinate were considered non-viable, since *Ephedra* spp. lack seed dormancy [24]. All necessary permits were obtained for the described field studies.

### Lizard movements

During the morning of July 21^th^ of 2004, we installed six pit-fall traps evenly distributed across the upper part of the study area (distance between consecutive traps was c. 50 m). Traps were located under reproductive fruiting females of Ephedra and baited with tomato. We selected a single lizard from those captured in each trap, in order to ensure that marked lizards were not close neighbours.

Six lizards (one from each trap) were tagged with radio-transmitters (Biotrack, Dorset, UK; weight = 0.35 g, expected life-span = 7–10 days) attached dorsally (between both shoulders) to the lizard skin by means of glue (SkinBond, Smith & Nephew United Inc., Largo, Florida, USA). Although home-range characteristics could differ between sexes [25], we only used adult males to ensure that radio-transmitters weighed less than 5% of the body weight (in our study population, virtually all females weight less than 8 grams).

Successive locations of radio-tagged lizards were determined from eight tracking stations, previously set and located with GPS and examined every 30–60 minutes. Tracking stations were spaced 10 to 240 m apart and located along a dirt track that delimited the upper side of our study site, in order to avoid disturbing the behaviour and activity of tracked lizards. From the first day after capture and until we started to lose their signal, we tracked the bearings of lizard tags using coordinated readings from two radio-receptors TR-4 with hand-held ‘H’ antennas (Telonics, Mesa, USA). Bearings were taken throughout the day (9:30 to 20:00), except during the inactivity period caused by high temperatures (early afternoon, between 14–16 hours). Bearings were translated into location points of radio-tagged lizards (locations, hereafter) using the best-biangulation method of the LOAS® software (Ecological Software Solutions). For each location, we calculated the net displacement, that is, the net distance to the starting point of the displacement track (i.e., the *Ephedra* plants where lizards were captured).

### Microhabitat selection

We complemented the radio-tracking data with observations of microhabitat preference and behaviour of lizards, based on visual surveys. To avoid interferences with the activity of the radio-tagged individuals, we measured lizard activity in the upper limit of our study site. During the morning (9:30 to 13:00, the period of maximum activity of lizards in the study area), we performed five daily surveys at each of five fixed stations (separated by c. 30 m). At each survey, we randomised the order at which stations were surveyed. At each station, we surveyed during five minutes (using binoculars whenever necessary) all lizards situated within a radius of approx. 10 m and recorded the habitat (sclerophylous shrub vs. *Ephedra* vs. open habitat) in which they were spotted. We also registered the behaviour of each individual, in the following categories: (a) moving, (b) feeding (counting the number of *Ephedra* fruits ingested whenever possible) and (c) passive activities. The last category included all behaviours (basking, guarding, resting, thermo-regulating and interacting with other individuals) of no direct relevance for the seed dispersal process. Total census time was 550 minutes. Since the visual-survey area differed from the whole study area in shrub cover (χ^2^_2_ = 6.5, *p* = 0.039; see also Fig. S1), microhabitat preferences were estimated against the relative cover of the three microhabitat types at the surveyed area (an 80-m strip in the upper limit of our study area).

It is important to note several limitations of our survey method. Firstly, because lizards were not trapped, they were neither tagged for individual identification nor reliably sexed. Hence, complete independence of data is not guaranteed (i.e., some individuals may have been censused twice, although the probability is reasonably low given the high abundance of lizards in the area; Santamaría unpubl. data) and we were not able to test for behavioural differences between sexes. Secondly, lizard detectability may have been lower under shrubs, as compared to open areas. Though we were aware of this possibility, and paid particular attention when surveying this habitat type, habitat preference for shrub-covered areas must be regarded as potentially under-estimated.

### Data analyses

All statistical analyses were performed using Generalized Linear (Mixed) Models (GLMs). For each analysis, we fitted the full model (as described below) and all its potential subsets of factors and covariates. We selected the ‘best model’ as the one with the lowest AICc (corrected Akaike Information Criterion). All environmental variables were standardized in order to get comparable coefficients independent of the measurement unit (i.e., we subtracted the mean from each variable's values and divided the result by the standard deviation). Unless otherwise indicated, average values are reported as mean ± standard error throughout the text. Owing the limited power of certain analyses, which are based on small sample sizes, we also report ‘marginally significant’ values (0.05<*p*<0.10) and interpret them to indicative inconclusive results (i.e., we can neither reject nor accept the null hypothesis).

**Gut-passage time and germination of seeds:** Gut-passage time of defecated seeds was fitted to a gamma error distribution and a log link function, with sex as fixed factor, mother-plant and lizard as random factors, and seed weight as continuous covariate. Seed germination was fitted to a binomial error distribution and a logit link function, with mother-plant as random factor, ‘treatment’ (defecated vs. non-ingested seeds) as a fixed factor, seed weight as continuous covariate and the interaction between treatment and seed weight. GLMs were fitted to data with the GLIMMIX procedure of the SAS statistical package [26].

**Habitat structure and lizard habitat preferences:** We approached the study of lizard habitat preferences from a multi-scale point of view, assuming that lizard locations were the result of various selection processes operating at three different scales. At the largest scale, the specific size and shape of the home-range of each individual lizard was hypothesized to depend on the habitat characteristics of the grid-cells included in such home-range, relative to the characteristics of those in its immediate neighbourhood (see below). At an intermediate scale (i.e., within home-range), each lizard was hypothesized to select for areas of suitable habitat within its home-range – i.e., to be preferentially found at grid-cells characterized by certain habitat features. Finally, at the smallest scale (i.e., within each grid-cell), lizards were hypothesize to spend more time within favourable microhabitats. Note that the grain of the three scales is imposed by the resolution of our field methodology, rather than by a pre-defined decision. Hence, analyses at the ‘home-range’ and ‘within home-range’ scales was based on radio-tracking data (number of locations per grid-cell), while analysis at the ‘microhabitat’ scale (within grid-cells) was based on data from the visual surveys.

We defined the home-range of each lizard as the area corresponding to the 95 percentile of the use density function, as estimated by a kernel home-range analysis (Fig. S2). To analyze habitat preference at the home-range scale, we tested which environmental variables determined the probability of a grid-cell to be included in the home-range of each lizard. With this aim, we first defined the area accessible to each individual lizard as that within a circle (centred at the home-range centroid) with radius equal to the maximum radius of its home-range. Then, each grid-cell within such ‘accessible area’ received a value of one if it was included in the home-range (i.e., if at least 20% of the cell's area was included within it) and a value or zero otherwise. At the within home-range scale, we assigned a value of one (presence) to each grid-cell of the focal home-range that included at least one lizard radio-location, and a value of zero (absence) otherwise. These values were respectively fitted to GLMs models based on measured environmental variables (see Fig. S2 for additional details).

Prior to fitting these models, we discarded highly correlated environmental (independent) variables to prevent problems of multicollinearity [27]. Whenever two variables were highly correlated (|*r*|>0.70), we selected one of them according to their capacity to explain the dependent variables in univariate GLMs (see Text S2 for details). Subsequently, we analysed the spatial aggregation of the environmental variables selected for the analyses, using Mantel correlograms at different distance-lags (*vegan* library within the R environment) [28].

Models explaining habitat preference at the home-range and within home-range scales were fitted using binomial error distributions and logit link-functions (*glmmML* library within the R environment) [28], and included the environmental covariates plus the random factor ‘individual lizard’. Residuals from the model with the lowest AICc value were checked for spatial autocorrelation by means of Mantel correlograms. If significant, we re-analysed the data using different spatial-correlation structures (i.e., linear, exponential, gaussian, ratio and spherical), using the *glmmPQL* library of R [28], and evaluated whether they improved the model's fit, residual distribution and predictive capacity. Finally, we assessed the performance of the selected model by means of the Area Under the ROC curve (a statistic based on the relationship between the true positive rate and the false positive rate when the discrimination threshold is varied, AUC hereafter; see also Fig. S4) [29], obtained with the *PresenceAbsence* library of R [28].

We validated our predictions of lizard habitat preferences using the *k*-fold cross-validation, a re-sampling approach to assess the robustness of measures with small datasets [30]: the dataset was divided into six independent elements (one per individual lizard) and, in six different runs, we computed the parameters estimates in five elements and validated them using the one element that had been left out.

Microhabitat preference was estimated by fitting the result of the visual surveys (proportion of sightings per habitat type) to a GLM with habitat, behaviour (moving, feeding or passive activities), day and their two-way interactions as fixed factors, site as random factor, a Poisson error distribution and a log link function.

**Figure 1:**
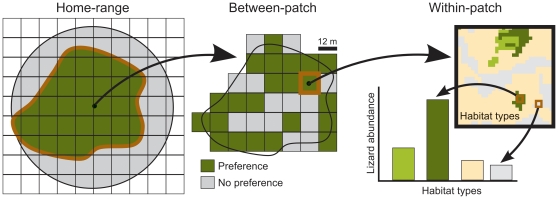
Conceptual diagram showing the three hierarchical scales of habitat preference by lizards. Home-range preferences, derived from the characteristics of the grid-cells included in the predicted home-range (dark green) and those in the area accessible to lizards (circle enclosing light-grey and dark-green areas), are determined broad-scale habitat features (c. 2.5–10*10^3^ m^2^). Within home-range preferences, defined as the presence (dark green) vs. absence (light grey) of telemetry locations in the grid-cells included within each home-range, reflect the preferential use of certain habitats within each home-range. Microhabitat preferences, based on surveys of lizard abundance at each habitat type, reflect the uneven use of space within each grid-cell. Grid-cells represent the spatial unit (‘grain’) of observation within our study area.

**Simulation of seed rain:** As indicated in the introduction and illustrated in Fig. 1, we assumed that lizard habitat preferences can be decomposed into three spatial scales (home-range, within home-range and microhabitat). Such preferences operate at hierarchical and increasingly smaller spatial scales: (1) ‘home-range’ preferences reflect habitat selection processes underlying the spatial configuration of each home-range within the landscape, (2) ‘within home-range’ preferences reflect habitat selection processes underlying the uneven use of space within each home-range, and (3) ‘microhabitat’ preferences reflect microhabitat selection processes underlying the uneven use of space at small scale (i.e., within grid-cells).

In order to simulate *Ephedra* seed rain, and because seed retention time and lizard displacement proved to have limited importance (see *Results*), we assumed that lizard habitat preferences at the three hierarchical spatial scales can be used as a *proxy* for the probability of deposition of ingested seeds. Based on this assumption, our modelling exercise aimed at evaluating the contribution of habitat preferences operating at these scales, both separately or in combination, to the spatial patterns of seed deposition (i.e., seed rain) by lizards. For this purpose, we used eight dispersal scenarios, which include a ‘random’ scenario (i.e., without habitat preference), three ‘single-scale’ scenarios (in which lizard habitat preference operates at a single scale: home-range vs. within home-range vs. microhabitat), three ‘double scale scenarios’ (in which lizard habitat preference operates at a combination of two scales: ‘home-range+within home-range’, ‘home-range+microhabitat’, and’ within home-range+microhabitat’) and a ‘three scales’ scenario (in which lizard habitat preference operates simultaneously at the three spatial scales; see also Text S3). We analysed the spatial aggregation of the resulting seed rain using Mantel correlograms at different distance-lags (as above) and used partial Mantel tests (*ecodist* library) [28] to perform pair-wise comparisons between the ‘random’ scenario and all other scenarios.

## Results

### Habitat structure

Open habitat was the most abundant habitat in the study site (60.8%), followed by rocks (29.0%), sclerophylous shrubs (8.2%) and *Ephedra* shrubs (1.9%). Shrub cover (sclerophylous+*Ephedra* shrubs, hereafter) was highly fragmented and heterogeneous, showing a declining gradient of patch size and cover from NW to SE (Fig. S1). After discarding the environmental variables with high inter-correlations (|*r*|<0.7) and low explanatory power (high univariate AICc, as compared to those of the selected variables; see Text S2), we retained five variables: slope, proportion of shrubs, proportion of rocks, number of shrub fragments and nearest-neighbour distance between shrub fragments.

Analyses of spatial autocorrelation revealed that the spatial structure of the habitat varied among environmental variables. Whereas the number of shrub fragments was aggregated at distances below 30 m, slope and shrub aggregation (nearest-neighbour distance between shrub fragments) did so at distances below 60 m, and the proportion of rocks and shrubs at less than 70 m (see Fig. S3).

### Seed retention time and lizard movement

**Figure 2:**
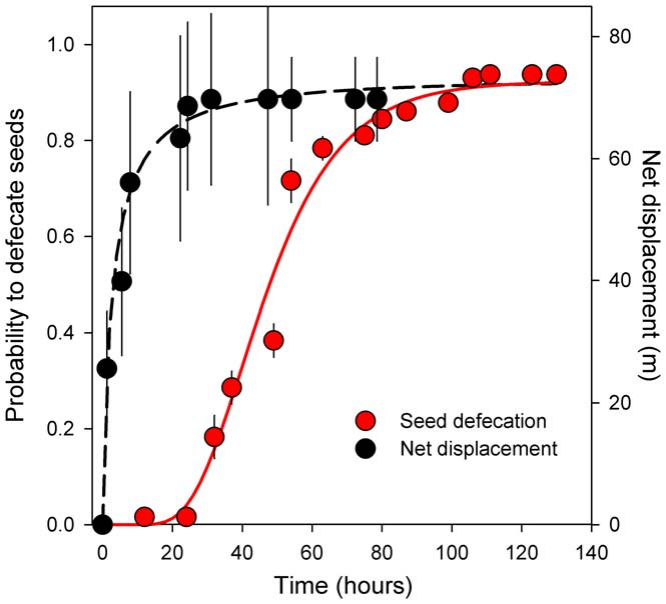
Cumulative frequency of defecation of *Ephedra* seeds ingested by lizards over time (i.e., proportion of ingested seeds defecated during such time interval; left axis), and net displacement (i.e., maximum net distance from the last relocation to the release location) of radio-tagged lizards over time (right axis). Lines represent accumulative log-normal fits. Symbols represent daily averages (±SE) each two hours of both variables (see material & methods); note, however, that fits were based on all measured values (not shown for clarity).

Retention time experiments showed that most seeds were defecated in the second and third day after ingestion (Fig. 2). Seed retention time did not vary with seed weight (F_1_,_74_ = 0.02, *p* = 0.884) or lizard sex (not included in the best model). Overall, ingested and non-ingested seeds showed similar (F_1_,_12_ = 1.03, *p* = 0.330) and fairly high (c. 80%) germination rates. However, germination probability decreased with increasing retention time and decreasing seed weight (log*_e_*(*p*/1−*p*) = −0.032*RT+0.290*SW+2.90; F_1_,_73_ = 5.53, *p* = 0.021 and F_1_,_73_ = 5.53, *p* = 0.021, respectively) and did not differ between sexes (F_1_,_73_ = 2.20, *p* = 0.142). Lizard net displacement saturated rapidly, reaching a maximum of approx. 72 m after 24 hours – i.e., before the peak of seed defecation (Fig. 2).

### Determinants of lizard habitat preferences

At the home-range scale, slope and the proportion of shrub cover were significantly associated with lizard habitat preferences (Table 1, Table S1). Lizards preferred flatter areas covered by shrubs (negative effect of slope, positive effect of shrub cover; Table 1). Within home-ranges, no environmental variable was significantly associated with lizard habitat preference, although shrub cover showed a marginally-significant, positive effect (Table 1). We obtained AUC values of 0.73±0.03 and 0.75±0.04 (Fig. S4) for the models at the home-range and within home-range scales, respectively, which indicates moderately good model performances. Predictive capacity, as estimated by *k*-fold cross-validation, was moderately good for the home-range (0.71±0.03) and poor for the within home-range (0.61±0.05) models.

**Table 1:** Results of Generalized Linear (Mixed) Model of (a) home-range, and (b) between-patch habitat preferences of lizards.

a) Home-range habitat preferences			
Intercept	−0.079±0.201	−0.39	0.694
Slope	−0.529±0.138	−3.84	<0.001
Shrub cover (%)	0.666±0.198	3.36	<0.001
Rock cover (%)	0.218±0.150	1.60	0.109
b) Between-patch habitat preferences			
Intercept	−0.652±0.394	−1.66	0.098
Shrub cover (%)	0.308±0.174	1.77	0.077

Only variable estimates (mean ± SE) from the model with lowest AICc score are shown. In all models, individual was included as a random factor, and environmental variables were standardized.

At both scales, the spatial autocorrelation of residuals was either weak (home-range: Mantel r = 0.105, *p* = 0.001) or non-significant (between-patch: Mantel r = 0.037, *p* = 0.199). At home-range scale, both raw data and best-model residuals showed significant spatial autocorrelation only at small distances (<30 m; Fig. S5). We inspected alternative spatial covariance structures for both models but, given that they did not improve the models' predictive capacity, we decided to retain the models without spatial covariance (see Table S2).

**Figure 3:**
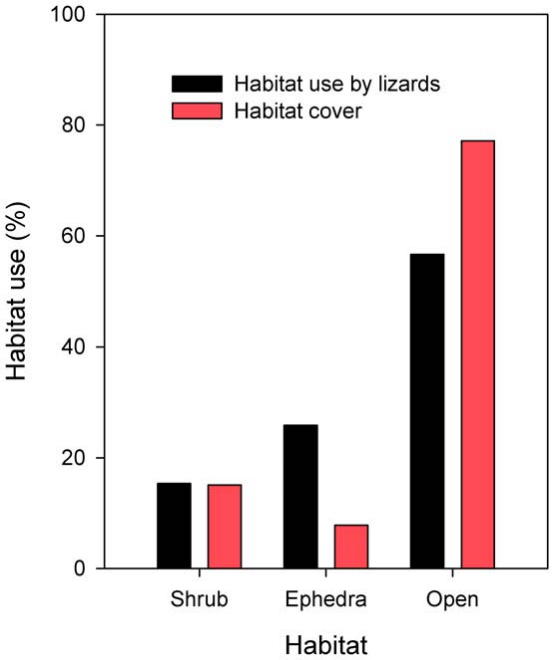
Habitat preference by lizards and habitat cover in the survey area at Dragonera Islet. Habitat preference by lizards (bars in black) was based on the proportion of visual censuses (*n* = 506 lizards) whereas habitat cover (bars in red) was calculated from a habitat map which only covers the area of visual censuses.

At microhabitat scale, we observed lizards more often in open habitat than under *Ephedra* or sclerophylous shrubs (Fig. 3). However, these proportions departed significantly from the null expectation of proportionality to relative habitat availability (χ^2^_2_ = 78.4, *p*<0.001): lizards were observed three times more often under *Ephedra* shrubs and 25% less often in open areas (Fig. 3). In addition, their behaviour varied significantly among habitats (habitat×behaviour: χ^2^_8_ = 20.7, *p* = 0.008): while most lizards observed in open habitats were moving (68%), many of those observed under *Ephedra* shrubs were feeding (30%) and consumed, on average, 18.5 fruits per hour (*n* = 18).

### Simulated seed rain

**Figure 4:**
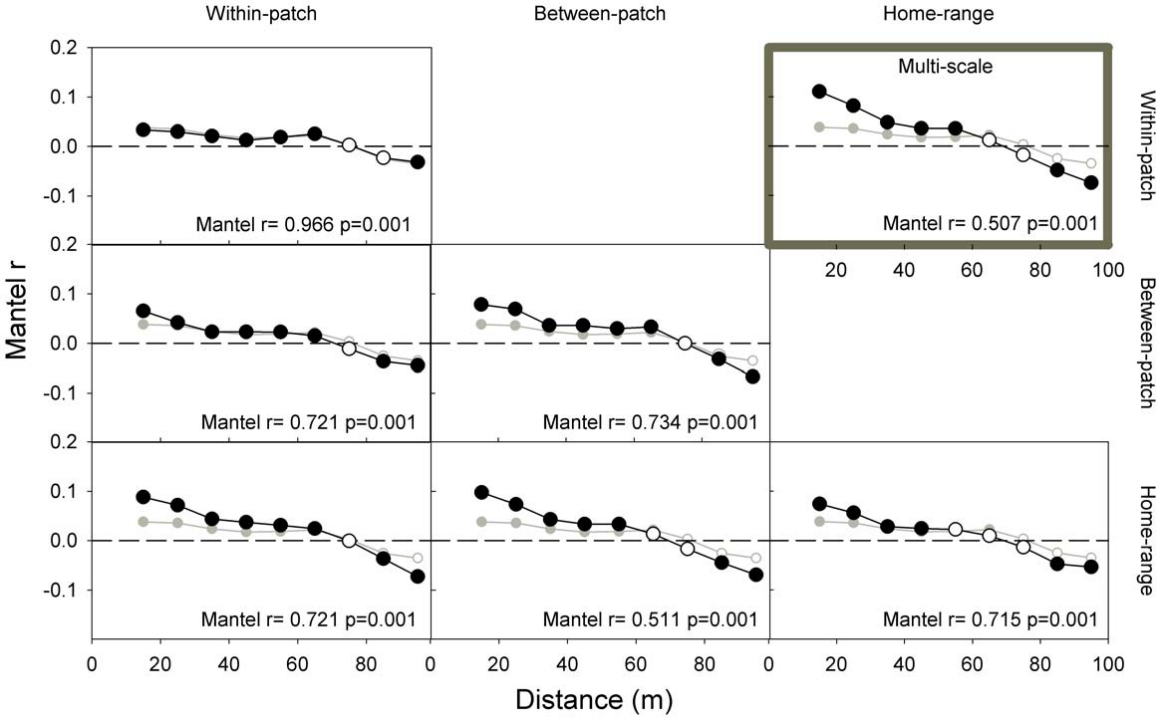
Mantel correlograms of simulated seed rain (seed dispersal probability per grid-cell) across the study-area lattice, using different habitat-dependent scenarios: one ‘random’ scenario, three ‘single-scale’ scenarios (home-range, within home-range and microhabitat preferences; diagonal panels) and their two-scales (‘home-range+within home-range’, etc.; lower-left panels) and three-scales (upper-right panel) combinations. At each distance, filled symbols indicate significant autocorrelations (Mantel correlation at a given distance matrix after sequential Bonferroni corrections), empty symbols non-significant ones. The ‘random’ scenario is depicted in grey and followed the same conventions as habitat-preference scenarios. At each panel, we also show the results of a partial-Mantel test comparing the similarity of the corresponding habitat-dependent scenario with the ‘random’ one (i.e., higher Mantel-r values indicate higher correlation between both scenarios, *p*<0.05 a significant departure from the null hypothesis of non-correlation). See Text S3 for further details.

Simulated seed rains showed, under all scenarios, comparable spatial structures (Fig. 4): non-significant autocorrelations at medium-large distances (50–80 m); significant, negative autocorrelations at larger (80–100 m) distances; and significant, positive autocorrelations at smaller (10–50 m) distances. However, autocorrelation strength increased considerably for the two scenarios that included, simultaneously, lizard preferences at two largest scales (‘home-range+within home-range’ and ‘three scales’). These two scenarios also showed a weaker (though still significant) pair-wise similarity with the ‘random’ scenario (Mantel r = 0.51) than all other scenarios (Mantel r>0.71), indicating a stronger discrepancy between their seed rain and that predicted in the absence of lizard habitat preferences.

## Discussion

In our study system, disperser behaviour (home-range characteristics and habitat preference) rather than physiology (gut passage time of seeds) determines the seed dispersal process – similar to what has been reported for the Balearic lizard *P. lilfordi* and the endemic shrub *Daphne rodriguezii* [9]. Since net displacement of *P. lilfordi* saturated earlier than the gut-passage time of seeds (c. 24 hours) and low germinability associated to prolonged seed retention times further restricted the likelihood of successful long-dispersal events, seed rain are chiefly determined by the home-range characteristics and habitat preferences of individual lizards.

These two determinants of *Ephedra* seed shadows were, in turn, influenced by habitat heterogeneity. The descriptors of landscape structure included in this study are likely surrogates of the multi-scale determinants of lizard habitat preference: food resources [31]–[32], refuges against predation [33]–[34] and thermoregulation sites [35]–[36]. At our study site, shrub cover influenced lizard activity at the three scales of analysis: lizards located their home-ranges preferentially in areas with abundant shrub cover (home-range scale), were found more often in those parts of their home-ranges with abundant shrub cover (within home-range scale) and used shrubs in a proportion that doubled their relative abundance within our observation area (microhabitat scale). Shrub cover provides an effective refuge against the main predators of Balearic lizards at Dragonera Islet (European kestrels, *Falco tinnunculus* and, occasionally, yellow-legged gulls *Larus michahellis*) [37] and, because *Ephedra* (which represents a prime food resource for lizards during masting, see above) and other sclerophylous shrubs tend to be aggregated in our study area (Fig. S1 and S6), it is reasonable to conclude that shrubs patches offer an optimal combination of food and refuge. In addition, the highly-significant effect of slope on home-ranges probably reflects costs of locomotion, while the marginally-significant effect of rock cover may indicate that, in areas offering high shrub cover, thermoregulation sites are limiting and therefore a preferred resource.

Hence, coming back to our original question: what are the potential effects of these habitat preferences on seed rain? Seed rain estimated under the different scenarios indicated that the influence of habitat structure on lizard habitat preferences may have cascading effects on the distribution of *Ephedra* seeds. Such effects required, however, the interplay of the two largest spatial scales to be detectable. As long as we left out of the simulations the combined effect of preferences at the home-range and within home-range scales, simulated seed rains were comparable to those of the ‘random’ scenario (without habitat preference) – i.e., they showed weak spatial structure, mostly at medium-small distances (<50 m). In contrast, the double- and triple-scale scenarios incorporating the join effect of home-range and within home-range preferences resulted in seed rains with considerably (two- to four-fold) higher spatial aggregation at the smallest (<30 m) and largest (>90 m) distances. Such increase in seed-rain aggregation reflects the interplay between the strong spatial structure of our study site and the responses of lizards to such heterogeneity (especially to shrub abundance). These results, which should be validated and tested in other locations and systems before any generalization is attempted, suggest that improving the accuracy of plant dispersal models (used e.g. to forecast plant invasion dynamics or responses to climate change) [38]–[40] will require a deeper understanding of the multi-scale factors regulating animal habitat preferences and activity [6].

In our study site, most environmental variables were structured at comparable distances (see Fig. S3), suggesting that a whole suite of habitat features could represent single, coherent sets of equivalent spatial information. From the point of view of the animal, the spatial autocorrelation of environmental variables implies spatial predictability of resources: for animals living in preferred, highly-visited areas, displacements beyond the threshold of the ‘autocorrelation signal’ (in our study system, 60–70 m) probably involves moving to lower-quality habitats (i.e., likely ‘sink habitats’) [41]. It is noteworthy that such threshold was found for the three variables influencing lizard habitat preference (slope and proportion of shrubs and rocks), the displacement distance measured for radio-tracked lizards (Fig. 2) and the positive-to-negative autocorrelation threshold obtained in the simulated seed rain. Such coincidence suggests that the spatial structure of the landscape, through its effects on habitat predictability, may determine the spatial-scale preference and activity of lizards, and leave a spatial imprint on the resulting seed rain [42].

Although our study system is a good example to test the effect of frugivore behaviour and physiology on seed rain (i.e., lizard is the most abundant frugivore in this population, and *Ephedra* fruits are an important fraction of the lizard diet in masting years), it also has important limitations that prevented us from validating our simulated seed rain with empirical data (presence and/or abundance of seeds and/or recruits; see Text S3). Hence, our use of habitat-preference scenarios is purely heuristic, aimed at providing insight on the potential consequences of incorporating disperser behaviour and multiple spatial scales in seed dispersal scenarios. Given that these potential consequences proved to be considerable, our future work will aim at obtaining data to refine, calibrate and validate these models for the *Ephedra*-lizard study system (e.g., using molecular markers).

In summary, our study shows that habitat preference may be a key determinant of seed rains generated by the frugivorous lizard *P. lilfordi*. These preferences arose at several spatial scales and interacted with the habitat's spatial heterogeneity to leave a discernible imprint in the spatial aggregation of deposited seeds: multi-scale scenarios incorporating disperser preferences at the two largest scales (home-range and within home-range) generated seed rains considerably more aggregated than those of the null, random-dispersal scenario. Further studies should examine whether this effect can be generalized to other sites and dispersal systems. Future research could aim at assessing the spatial scales at which habitat preferences of dispersers shape plant distribution [42]; whether habitat structure can be used as a *proxy* for disperser activity and the structure of the resulting seed rain [10]; whether concordant or discordant effects of landscape structure on seed and seedling fate may favour or disfavour specific sites [13], [14]; or the effect of inter-annual variation in fruit production (e.g., masting behaviour) on disperser activity and seed-shadow characteristics [43].

## Supporting Information

Figure S1 **Study site at Dragonera Islet.**
(DOC)

Figure S2 **Measures of lizard activity at the home-range and within home-range scales.**
(DOC)

Figure S3 **Mantel correlograms of environmental variables in the study site slope, shrub cover (%), rock cover (%), number of shrub fragments, and nearest-neighbour distance between shrub fragments.**
(DOC)

Figure S4 **Receiver Operator Characteristic (ROC) curves showing the intrinsic qualities of the predictions of Generalized Linear (Mixed) Models estimating lizard habitat preference at home-range and within home-range scales.**
(DOC)

Figure S5 **Spatial autocorrelation of dependent variables and model residuals Mantel correlograms for the dependent variables (presence/absence of telemetry locations) and the residuals of the best Generalized Linear (Mixed) Models fitted to them, at two habitat-preference scales: home-range and within home-range.**
(DOC)

Text S1 **Fragmentation indexes.** We calculated fragmentation indexes using the software FRAGSTAT.
(DOC)

Text S2 **Selection of independent variables for the analyses of lizard habitat preferences (‘home-range’ and ‘within home-range’ scales).**
(DOC)

Text S3 **Simulated seed shadows.**
(DOC)

Table S1 **Results of Generalized Linear (Mixed) Models predicting lizard habitat preference at home-range and within home-range.**
(DOC)

Table S2 **Effect of covariance structure on the spatial autocorrelation of model residuals.**
(DOC)
