# Supplementary material for: Effects of Frugivore Preferences and Habitat Heterogeneity on Seed Rain: A Multi-Scale Analysis
Source: PLoS One. 2012 Mar 16;7(3):e33246. doi: 10.1371/journal.pone.0033246 (PMC3306386; doi:10.1371/journal.pone.0033246)

**Fig. S1 - Study site at Dragonera Islet**

Different colours indicate the four habitat categories considered in the study site (pixel size: 0.16 m^2^). Red lines indicate the 12 x 12 m grid-cells used for analyses. Grid-cell size was chosen to coincide with the telemetry location error of radio-tracked lizards (i.e., 11.6 ± 0.3 m). Contour lines represent 5-m isolines derived from a Digital Elevation Model (DEM).

We entered data on environmental variables of the study site in a GIS platform (ArcGIS 9.2, ESRI® ArcMap^TM^ 9.2) that included: (a) a Digital Elevation Model (DEM), based on 1:1000 cartography, and (b) a habitat map, derived from an aerial ortho-photograph using a supervised classification, with thematic categories adjusted to match our field observations. Habitat categories were: ‘Ephedra shrub’, ‘sclerophyllous shrub’, ‘rock’ and ‘bare soil’. The two shrub categories were highly correlated (Spearman rank correlation: r=0.953, *p*<0.001); hence, we pooled them into a single one (‘shrub’).


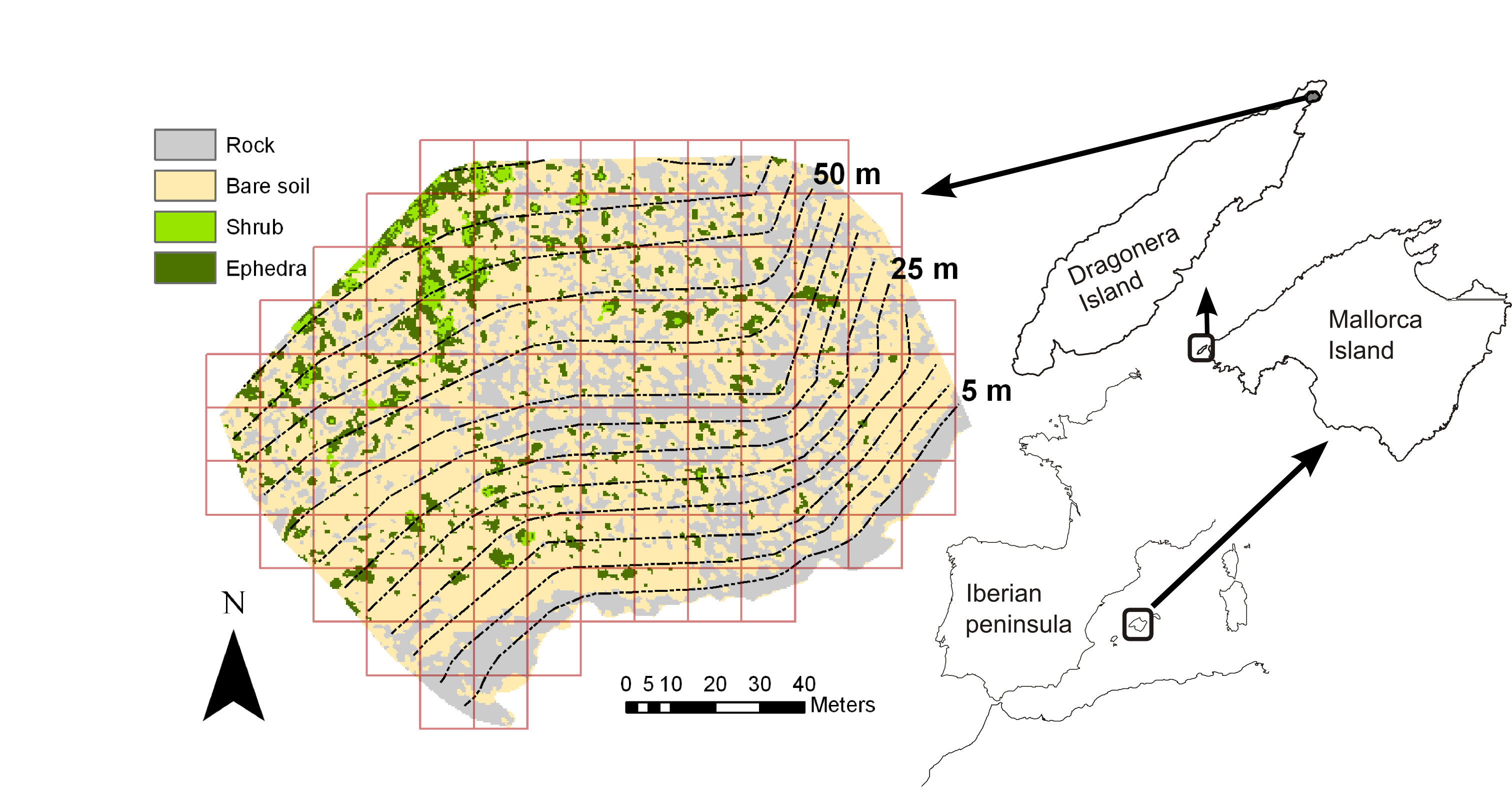

Supplement: Figure S1 — Study site at Dragonera Islet. (DOC) [file pone.0033246.s001.doc]
