# Supplementary material for: Effects of Frugivore Preferences and Habitat Heterogeneity on Seed Rain: A Multi-Scale Analysis
Source: PLoS One. 2012 Mar 16;7(3):e33246. doi: 10.1371/journal.pone.0033246 (PMC3306386; doi:10.1371/journal.pone.0033246)
Supplement: Figure S2 — Measures of lizard activity at the home-range and within home-range scales. (DOC) [file pone.0033246.s002.doc]

**Fig. S2 – Measures of lizard activity at the home-range and within home-range scales**

The analysis of the environmental determinants of lizard home-ranges began by defining the area available for each individual lizard. Such ‘available area’ was defined by a circle around the centroid of each home-range, with a radius equal to the maximum distance between the home-range boundary and such centroid. We defined the home-range of a lizard (light green polygons, average area = 4,389.5 m^2^) as the 95 percentile of a use-density function estimated by means of an adaptive-kernel, obtained using the least-squares cross-validation method to choose the smoothing or ‘window size’ parameter [1]. Use-density functions were estimated using the extension ‘Home Range Tools’ for ArcGis 9.0 [2]. Then, grid-cells (our basic spatial unit of landscape characterization; see Fig. S1) were classified as ‘home-range presences’ (and assigned a value or 1) if at least 20% of their surface overlapped with the estimated home-range (red *grid-cells*). ‘Home-range absences’ (black *grid-cells*) were defined as those grid-cells within the ‘accessible area’ of the focal lizard (i.e., the dark green polygon) but outside the lizard’s home-range. After repeating this process for all individual lizards, we related the environmental variables to the proportion of grid-cells classified as presences or an absences (see Data analyses).

Numbers within grid-cells represent the number of lizard radio-tracking locations recorded in that particular cell (see *Material and Methods*). Within a given home-range, each grid-cell was classified as a presence if it included at least one radio-tracking location (grid-cell values greater than zero in the figure), and as a absence otherwise. The probability of a cell containing at least one radio-location was then modelled as a function of our environmental variables (see *Data analyses*).

Grid-cells with red circles indicate the capture point of each radio-tracked lizard.


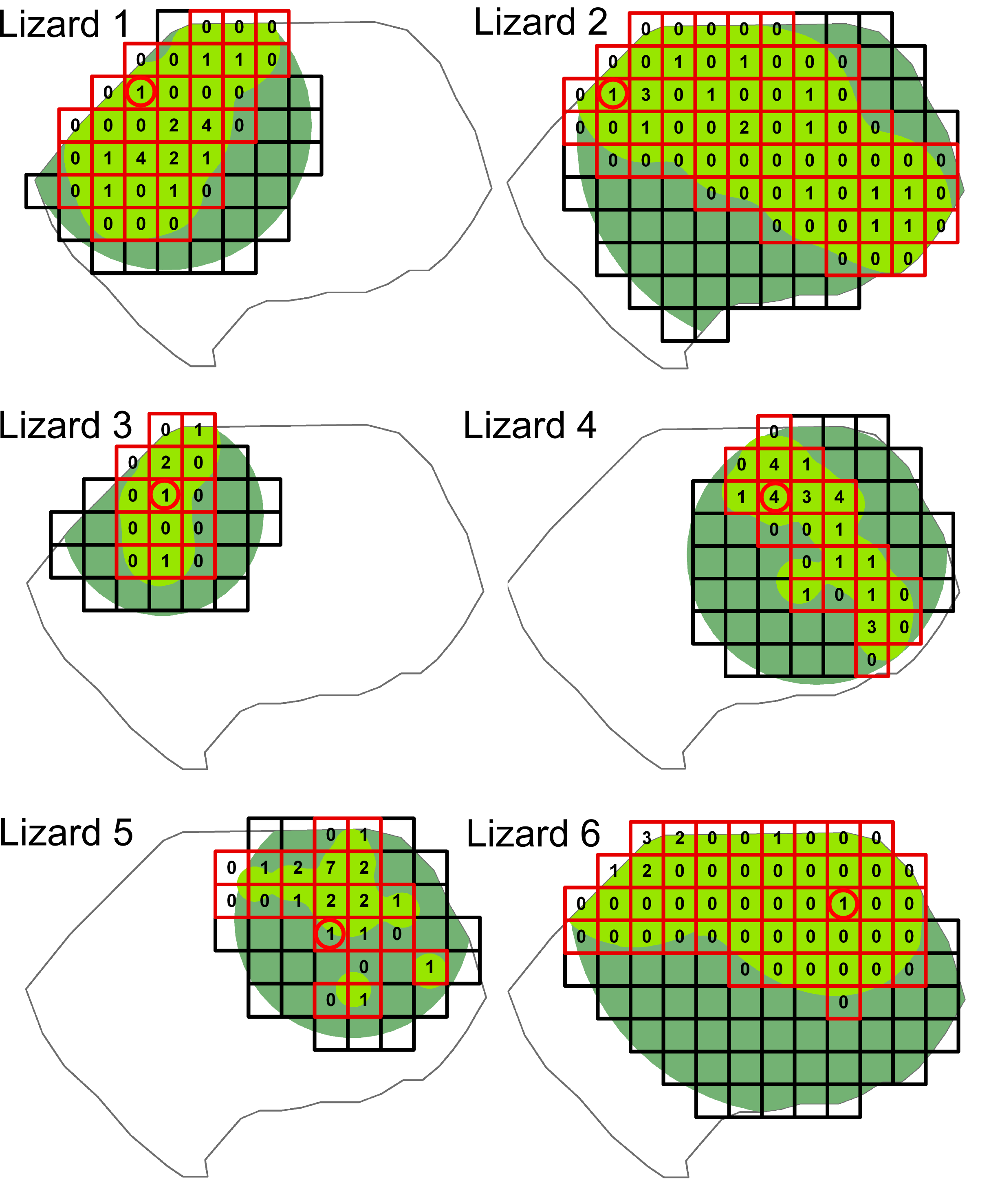


**References**

1. Worton BJ (1989) Kernel methods for estimating the utilization distribution in home-range studies. Ecology 70:164-168.
2. Rodgers AR, Carr AP, Smith L, Kie JG (2005) HRT: Home range tools for ArcGIS®. Thunder Bay, Ontario, Canada, Ontario Ministry of Natural Resources, Centre for Northern Forest Ecosystem Research.
