# Supplementary material for: Effects of Frugivore Preferences and Habitat Heterogeneity on Seed Rain: A Multi-Scale Analysis
Source: PLoS One. 2012 Mar 16;7(3):e33246. doi: 10.1371/journal.pone.0033246 (PMC3306386; doi:10.1371/journal.pone.0033246)
Supplement: Figure S3 — Mantel correlograms of environmental variables in the study site slope, shrub cover (%), rock cover (%), number of shrub fragments, and nearest-neighbour distance between shrub fragments. (DOC) [file pone.0033246.s003.doc]

**Fig. S3 - Mantel correlograms of environmental variables in the study site**

(a) slope, (b) shrub cover (%), (c) rock cover (%), (d) number of shrub fragments, and (e) nearest-neighbour distance between shrub fragments. Filled symbols indicate significant spatial autocorrelations, empty symbols non-significant ones. Tests of significance were based on 1000 permutations of rows and columns of the dissimilarity matrix. Significance of each distance class was corrected for multiple testing using the Bonferroni-Holm procedure. Tests were computed with the *vegan* library [1] of the R environment [2].

**References**

1. Oksanen J, Blanchet FG, Kindt R, Legendre P,, O'Hara RB, Simpson GL, Solymos P, Stevens MH Wagner H (2010). vegan: Community Ecology Package. R Packaged version 1.17-4. <http://CRAN.R-project.org/package=vegan>
2. R Development Core Team (2009) R: A language and environment for statistical computing. In: R Foundation for Statistical Computing (ed) Vienna, Austria
