# Supplementary material for: Effects of Frugivore Preferences and Habitat Heterogeneity on Seed Rain: A Multi-Scale Analysis
Source: PLoS One. 2012 Mar 16;7(3):e33246. doi: 10.1371/journal.pone.0033246 (PMC3306386; doi:10.1371/journal.pone.0033246)
Supplement: Figure S4 — Receiver Operator Characteristic (ROC) curves showing the intrinsic qualities of the predictions of Generalized Linear (Mixed) Models estimating lizard habitat preference at home-range and within home-range scales. (DOC) [file pone.0033246.s004.doc]

**Fig. S4 – Receiver Operator Characteristic (ROC)** **curves** showing the intrinsic qualities of the predictions of Generalized Linear (Mixed) Models estimating lizard habitat preference at home-range and within home-range (left and right panels, respectively) scales. For a binary classifier system, the quality of predictions depends on its ability to identify true positives (i.e., sensitivity = TP/(TP+FN); where TP = true positives and FN = false negatives; X-axis) and true negatives (i.e., specificity = TN/(TN+FP); where TN = true negatives and FP = false positives; Y-axis). The ROC curve is a graphical plot of the true positive rate (sensitivity) vs. the false positive rate (1 – specificity) as the discrimination threshold is varied. The Area Under the ROC Curve (AUC) provides a metric of the model’s predictive power [1], ranging from 0.5 (null predictive power) to 1.0 (perfect prediction). The 1:1 line represents the expected probabilities for randomly distributed presences and absences.


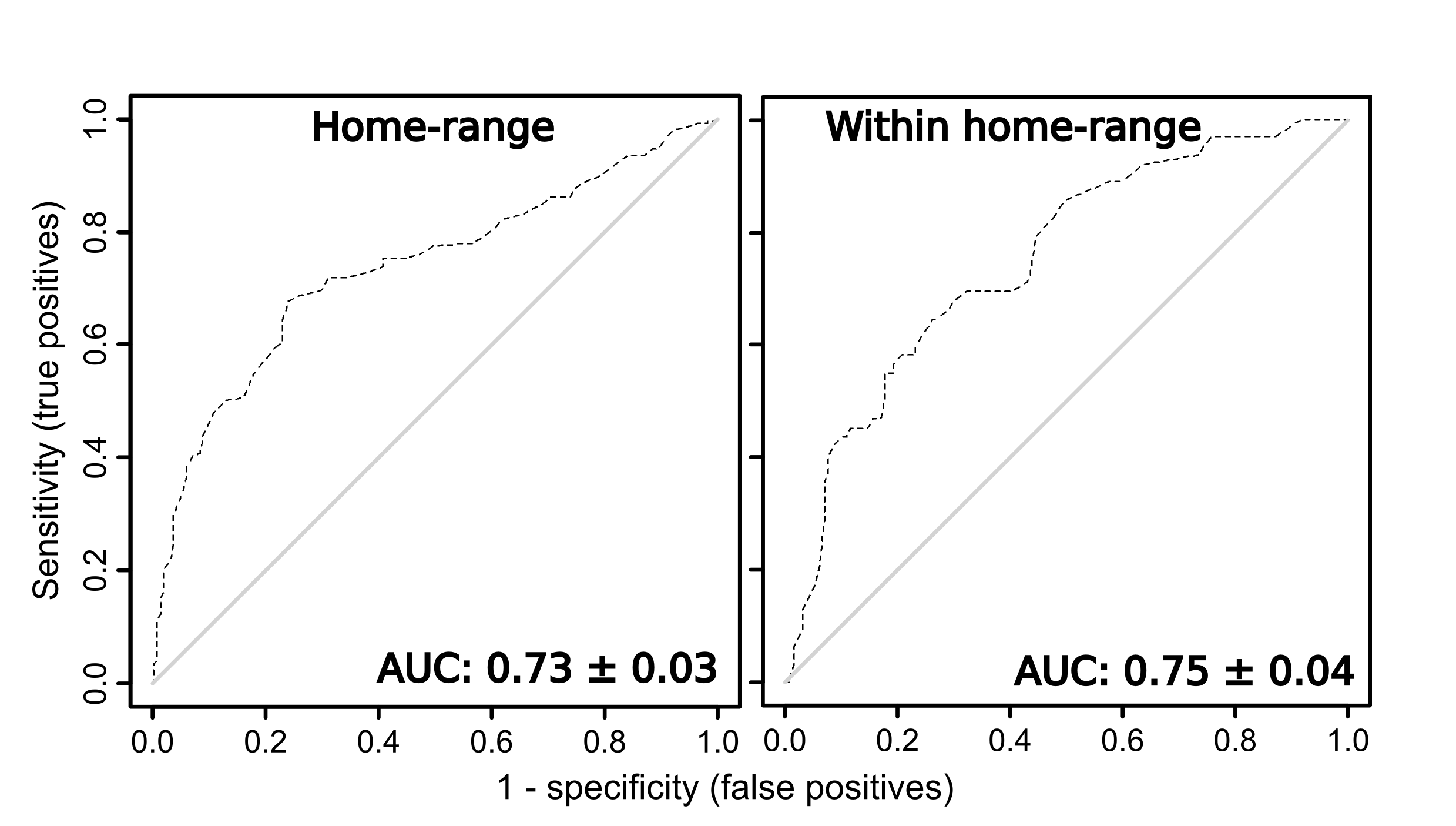


**References**

1. Boyce MS, Vernier PR, Nielsen SE, Schmiegelow FKA (2002) Evaluating resource selection functions. Ecological Modelling 157:281-300
