# Supplementary material for: Effects of Frugivore Preferences and Habitat Heterogeneity on Seed Rain: A Multi-Scale Analysis
Source: PLoS One. 2012 Mar 16;7(3):e33246. doi: 10.1371/journal.pone.0033246 (PMC3306386; doi:10.1371/journal.pone.0033246)
Supplement: Figure S5 — Spatial autocorrelation of dependent variables and model residuals Mantel correlograms for the dependent variables (presence/absence of telemetry locations) and the residuals of the best Generalized Linear (Mixed) Models fitted to them, at two habitat-preference scales: home-range and within home-range. (DOC) [file pone.0033246.s005.doc]

**Fig. S5 – Spatial autocorrelation of dependent variables and model residuals**

Mantel correlograms for the dependent variables (presence/absence of telemetry locations; upper panels) and the residuals of the best Generalized Linear (Mixed) Models fitted to them (lower panels), at two habitat-preference scales: home-range (left panels) and within home-range (right panels). Filled symbols indicate significant spatial autocorrelations (with sequential Bonferroni corrections), empty symbols non-significant ones.
