# Supplementary material for: Effects of Frugivore Preferences and Habitat Heterogeneity on Seed Rain: A Multi-Scale Analysis
Source: PLoS One. 2012 Mar 16;7(3):e33246. doi: 10.1371/journal.pone.0033246 (PMC3306386; doi:10.1371/journal.pone.0033246)
Supplement: Text S1 — Fragmentation indexes. We calculated fragmentation indexes using the software FRAGSTAT. (DOC) [file pone.0033246.s006.doc]

**Text S1 – Fragmentation indexes**

We calculated fragmentation indexes using the software FRAGSTAT [1]. This program computes several fragmentation statistics at the class and landscape level, quantifying landscape composition and configuration. All metrics were calculated exclusively for the ‘shrub’ category (i.e., sclerophillous shrub + *Ephedra* shrub); rock and open habitat were both included in the matrix surrounding shrub patches (see Fig. S1 for a definition of the four habitat categories). Metrics were calculated for each of the 110 cells defined by the lattice shown in Fig. S1. We selected four fragmentation indexes:

1. NP: Number of shrub patches (per grid-cell).
2. AREA: Mean shrub-patch area (in ha).
3. ENN: Euclidean distance (m) to the nearest-neighbouring patch, based on the shortest edge-to-edge distance within each grid-cell.
4. SHAPE: Patch perimeter divided by the minimum perimeter possible (i.e., that of a round patch of identical area), averaged among all shrub patches found within each grid-cell. SHAPE equals 1 for maximally compact (circular) patches, and increases without limit (i.e., SHAPE ≥ 1) as patch shape becomes more irregular.

Calculations were based on the script ‘FragStatsBatch for Arcgis 9’, designed by Brian Mitchell to extract FRAGSTAT metrics within the ArcMap^TM^ environment (see <http://www.umass.edu/landeco/research/fragstats/fragstats.html> for computation details).

**References**

1. McGarigal K, Cushman SA, Neel MC, Ene E. (2002) FRAGSTATS: Spatial pattern analysis program for categorical maps. University of Massachusetts, Amherst.
