# Supplementary material for: Effects of Frugivore Preferences and Habitat Heterogeneity on Seed Rain: A Multi-Scale Analysis
Source: PLoS One. 2012 Mar 16;7(3):e33246. doi: 10.1371/journal.pone.0033246 (PMC3306386; doi:10.1371/journal.pone.0033246)
Supplement: Text S2 — Selection of independent variables for the analyses of lizard habitat preferences (‘home-range’ and ‘within home-range’ scales). (DOC) [file pone.0033246.s007.doc]

**Text S2 – Selection of independent variables for the analyses of lizard habitat preferences (‘home-range’ and ‘within home-range’ scales)**

Before fitting Generalized Linear (Mixed) Models to lizard activity data at the ‘home-range’ and ‘within home-range’ scales, we identified and removed correlated variables to avoid biases caused by multicollinearity [1], as follows: We tested the cross-correlations between all environmental (independent) variables (Table S2.1), and created univariate models of each environmental variable with both dependent variables (i.e., ‘home-range’ and ‘within home-range’ presence/absence; Table S2.2). Whenever two or more independent variables were highly correlated (|*r*| > 0.70), we selected the one that performed as the best predictor based on such univariate models (i.e., the one with the lowest AICc score) - separately for each dependent variable [2, 3].

Table S2.1 - Pair-wise correlations between environmental variables. Lower-left and upper-right sections of the table respectively show two-way Spearman-rank correlations and their associated probability. Values in bold indicate high correlation (i.e., |*r*| > 0.70) between pairs of independent variables. Abbreviations: %Shrub = shrub cover (*Ephedra* + sclerophyllous shrub; see Fig. S1), as percentage. %Rock = rock cover, as percentage. NF = Number of shrub fragments. AREA = Area of shrub fragments (ha). ENN = Euclidean distance to nearest-neighbour shrub fragment. SHAPE = Shrub fragment shape (decreasing towards 1 in less irregular, more packed patches).

|  | Height | Slope | %Shrub | %Rock | NF | AREA | ENN | SHAPE |
| --- | --- | --- | --- | --- | --- | --- | --- | --- |
| Height |  | <0.001 | <0.001 | <0.05 | <0.001 | <0.001 | <0.001 | <0.001 |
| Slope | -0.584 |  | <0.001 | n.s. | <0.01 | <0.001 | <0.01 | <0.001 |
| %Shrub | 0.628 | -0.469 |  | <0.001 | <0.001 | <0.001 | <0.001 | <0.001 |
| %Rock | -0.221 | 0.145 | -0.580 |  |  |  |  |  |
| NF | 0.440 | -0.305 | 0.464 | -0.380 | 0.580 | <0.001 | <0.001 | <0.001 |
| AREA | 0.569 | -0.443 | 0.686 | -0.452 | 0.647 |  | <0.001 | <0.001 |
| ENN | -0.473 | 0.288 | -0.540 | 0.503 | **-0.817** | **-0.745** |  | <0.001 |
| SHAPE | 0.483 | -0.315 | 0.608 | -0.418 | 0.612 | **0.900** | **-0.768** |  |

Table S2.2 – AICc scores of univariate Generalized Linear Mixed Models of ‘home range’ and ‘within home-range’ presence/absence. In all models, lizard individual was included as random factor. Models were fitted to binomial error distributions and logit link-functions (*glmmML* library within the R environment) [4]. Abbreviations as in Table S2.1.

| Independent variables | d.f. | Home- range | Within home-range |
| --- | --- | --- | --- |
| Height | 4 | 700.8 | 258.3 |
| Slope | 4 | 782.1 | 259.1 |
| %Shrub | 4 | 795.5 | 254.5 |
| %Rock | 4 | 811.1 | 259.8 |
| NF | 4 | 803.2 | 258.3 |
| AREA | 4 | 785.9 | 256.1 |
| ENN | 4 | 787.0 | 258.1 |
| SHAPE | 4 | 779.0 | 256.1 |

**References**

1. Quinn GP, Keough MJ (2002) Experimental design and data analysis for biologists. Cambridge University Press, Melbourne.
2. Burnham KP, Anderson DR (2003) Model selection and multimodel inference. Springer-Verlag.
3. Tabachnick B, Fidell L (1996) Using Multivariate Statistics. New York: HarperCollins Publishers Inc. 880 p.
4. Broström G (2009). *glmmML*: Generalized linear models with clustering. R package version 0.81-6. http://CRAN.R-project.org/package=glmmML
