# Supplementary material for: Effects of Frugivore Preferences and Habitat Heterogeneity on Seed Rain: A Multi-Scale Analysis
Source: PLoS One. 2012 Mar 16;7(3):e33246. doi: 10.1371/journal.pone.0033246 (PMC3306386; doi:10.1371/journal.pone.0033246)
Supplement: Text S3 — Simulated seed shadows. (DOC) [file pone.0033246.s008.doc]

**Text S3 – Simulated seed shadows**

Our simulations assumed that the three hierarchical spatial scales at which we analyzed lizard habitat preference (home-range, within home-range and microhabitat) could be considered as a *proxy* of the probability of deposition of ingested seeds. Hence, the probability of seed deposition at a given grid-cell is proportional to the probability at which lizards visit it (as determined by the lizard’s preferences at home-range and within home-range scales) and, within that grid-cell, seed deposition at each different microhabitat is proportional to lizard microhabitat preferences.

We want to note that several limitations of our study system prevented us from obtaining reliable data for the validation of seed rain. In fact, the numerous difficulties involved in obtaining direct measurements of seed rain were our initial motivation to try to derive seed rain from simulations based on habitat preferences. First, measuring seed deposition by collecting either seeds or lizard scats proved to be extremely difficult and unreliable, owing to the specific nature of the terrain (a rugged, karstic landscape with numerous wholes and crevices, interspersed with small pockets of soil covered by shrubs, litter and dried herbaceous plants). Second, mapping seedlings was unfeasible owing to their scarcity, probably caused by low germination and establishment in all but exceptionally wet years (e.g., plants rarely recruit in experimental sowings; unpubl. data) and high rates of seedling predation by introduced European rabbits (unpubl. data). Third, mapping the distribution of juveniles was unreliable because *Ephedra* is a re-sprouting shrub and juveniles are not easily distinguished from re-sprouts. Finally, we did not validate our results with the abundance of adults because it was strongly correlated with shrub cover (see Fig. S1), an input variable in the seed-dispersal model. In consequence, the main objective of the model is purely heuristic, instead of predictive - i.e., it is exclusively aimed at exploring the consequences of disperser behaviour patterns on seed rain, not at predicting its specific shape.

Since we were interested in simulating *Ephedra* seed rain at landscape scale, the probability of deposition was evaluated in each of the 110 grid-cells included in the lattice defining our study area (Fig. S1). For each grid-cell, the probability of lizard visitation (hence, of seed deposition) was estimated as the multiplication of three probabilities, respectively representing lizard habitat preference at the ‘home-range’, ‘within home-range’ and ‘microhabitat’ scales. Thus, the probability of seed deposition in a given grid-cell and microhabitat type depends on three hierarchical processes: (1) the probability that such grid-cell is included in a lizard’s home-range; (2) the probability that such grid-cell is visited by the lizards inhabiting such home-ranges; and (3) the probability of lizard visitation to each of the two microhabitat types (i.e., its microhabitat preferences).

For each of these three scales, lizard habitat preference was either derived from the statistical models fitted to our field data (‘habitat preference’) or from a null-model (‘no habitat choice’, or ‘random preference’; see below for details). The combination of both possible models at the three scales of habitat preference resulted in eight seed-dispersal scenarios (Table S3.1, Fig. S3.1), which respectively correspond to:

(1) One null *random* *scenario*, which did not depend on habitat preferences at any of the three spatial scales (meaning that random preference operates at the three spatial scales).

(2) Three *single-scale habitat scenarios*, based on estimated lizard habitat preferences at either the home-range, within home-range or microhabitat scales. For each scenario, the two scales of habitat-preference that were not derived from the habitat-preference models were assigned at random.

(3) Three *double-scale habitat scenarios*, based on estimated habitat preferences at the three possible combinations of two of the scales of habitat preference listed above (i.e., home-range + within home-range, home-range + microhabitat, and within home-range + microhabitat). For each scenario, the scale of habitat-preference that was not derived from the habitat-preference models was assigned at random.

(4) One t*riple-scale habitat scenario*, based on habitat preferences estimated at each of the three spatial scales (i.e., home-range + within home-range + microhabitat).

Table S3.1 - Summary table of scenarios simulating the seed rain of *Ephedra fragilis* dispersed by Balearic lizards at Dragonera Islet. For each scenario and scale, we indicate whether the seed deposition at each grid-cell depended on lizard habitat preferences (*Habitat*) or were assigned at random (*Random*).

| Spatial scale: | Home-range | | Within home-range | | Microhabitat | |
| --- | --- | --- | --- | --- | --- | --- |
| Model scenarios | Habitat | Random | Habitat | Random | Habitat | Random |
| 1. Random (null model) |  | X |  | X |  | X |
| 1. Single-scale home range (HR) | X |  |  | X |  | X |
| 1. Single-scale between-patch (BP) |  | X | X |  |  | X |
| 1. Single-scale within-patch (WP) |  | X |  | X | X |  |
| 1. Double-scale ‘HR + BP’ | X |  | X |  |  | X |
| 1. Double-scale ‘HR + WP’ | X |  |  | X | X |  |
| 1. Double-scale ‘BP + WP’ |  | X | X |  | X |  |
| 1. Triplescale ‘HR + BP + WP‘ | X |  | X |  | X |  |

Deposition probabilities for each *i* grid-cell were calculated for each *j* spatial scales of preferences. These probabilities were calculated for the whole study-site lattice (*n_i_* = 110, based on (a) ‘habitat’ preferences, derived from statistical models fitted to our field data, and (b) ‘random’ preferences, generated from randomly-assigned probabilities:

1. Home–range scale: Under the ‘random’ scenario, each *i* grid-cell had randomly-assigned probabilities (*p_1i_*) to be included in a home-range; i.e., the probability of inclusion in a home-range was assigned to each grid-cell using a string of 110 random-generated numbers ranging from 0.0 to 1.0 (i.e., for each *i* grid-cell we draw a random number). For *Habitat* preferences, the probability that a given *i* grid-cell is included in a home-range depended on its slope (*Slope*), shrub cover (*%Shrub*) and rock cover (*%Rock*), according to the formula:

*p_1i_*=e-0.079-0.529**Slope[i]*+0.666*%*Shrub[i]*+0.218*%Rock[i]/

(1+e-0.079-0.529**Slope[i]*+0.666*%*Shrub[i]*+0.218*%Rock*[i]*) (1)

which was derived from the GLM estimates presented in Table 1 (see *Results*).

1. Within home-range scale: Under the ‘random’ scenario, all *i* grid-cell within a given home-range had randomly-assigned probabilities (*p_2i_)* to be visited; i.e., visitation probability for each *i* grid-cell was extracted from a string of 110 random-generated numbers. Under ‘habitat preference’, visitation probability of each *i* grid-cell included in a given home-range depended on its shrub cover (%*Shrub*), according to the formula:

*p_2i_*=e^-0.652 +0.308*%^*^Shrub[i]^*/(1+e^-0.652 +0.308*%^*^Shrub[i]^*) (2)

which was derived from the GLM estimates presented in Table 1 (see *Results*).

1. Microhabitat scale: Visitation probabilities of each microhabitat (open habitat vs. shrub) within each *i* grid-cell were directly proportional to their relative cover within such grid-cell; i.e., we assumed that lizards would visit both microhabitat types with identical probabilities. Under the ‘habitat preference’ model, the probability of lizard visitation in a given microhabitat resulted from the multiplication of its relative cover (as above) by the relative frequency of visitation of that microhabitat (*f_O_*=0.57 and *f_S_*=0.43 for open and shrub-covered areas, respectively; see also Fig. 3). The resulting probabilities added to re-calculate the probability (of seed deposition) per grid-cell and habitat type; hence:

*p_3i_* = *%Shrub[i]***f_S_* + *%Open[i]***f_O_* (3)

At each of the three scales, the probabilities of lizard preference were standardized to ensure that the visitation probability over the whole study area (which included 110 grid-cells) equalled 1.0. The final output of each scenario presented in Table S3.1 and Fig.S3.1 was, for *j* spatial scales and *i* grid-cell, the probability of lizard visitation to each grid-cell and microhabitat type, obtained by multiplying the probabilities obtained at each scale; hence:

*P_i_* = *p_1i_* * *p_2i_* * *p_3i_* (4)

Similar to the probabilities at each scale, the visitation probability also totalled 1.0 when added over the whole study area.


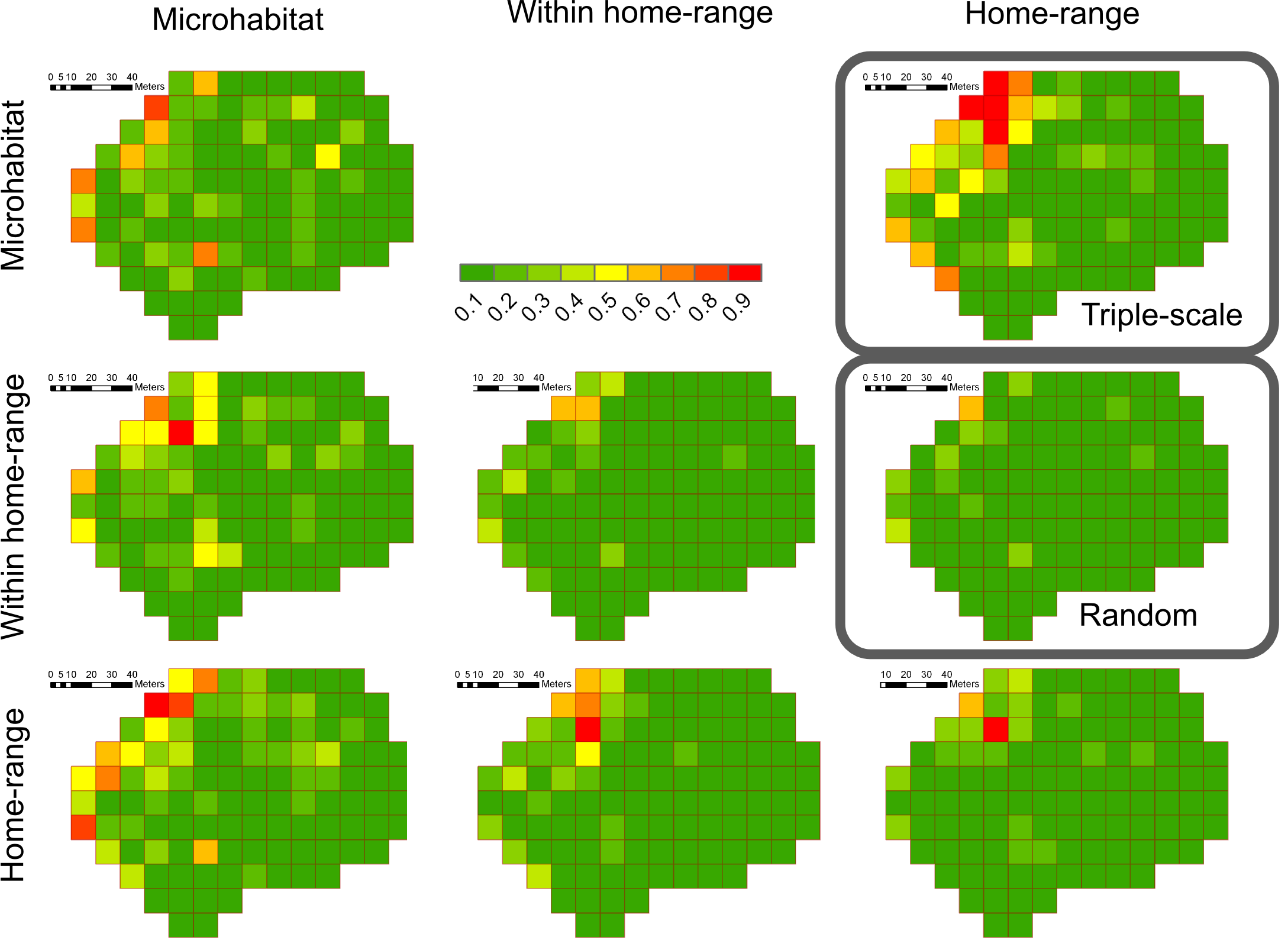


Fig. S3.1 – Results of eight scenarios of habitat preference simulating the seed rain (spatial patterns of seed deposition) of *Ephedra fragilis* dispersed by Balearic lizards in Dragonera Islet. The lattice representing our study area includes 110 grid-cells. Different panels summarize the results of a completely random scenario (‘Random’, middle-right panel), three single-scale scenarios (based on lizard habitat preferences at either the home-range, within home-range or microhabitat scales; diagonal panels, in which the scale of lizard habitat preference is indicated by the row or column headings), three double-scale scenarios (lower-left panels; the combination of scales is indicated by the row and column headings) and one triple-scale scenario (upper-right panel).
