# Supplementary material for: Effects of Frugivore Preferences and Habitat Heterogeneity on Seed Rain: A Multi-Scale Analysis
Source: PLoS One. 2012 Mar 16;7(3):e33246. doi: 10.1371/journal.pone.0033246 (PMC3306386; doi:10.1371/journal.pone.0033246)
Supplement: Table S1 — Results of Generalized Linear (Mixed) Models predicting lizard habitat preference at home-range and within home-range. (DOC) [file pone.0033246.s009.doc]

**Table S1 - Results of Generalized Linear (Mixed) Models predicting lizard habitat preference**

at (a) home-range, and (b) within home-range. Results shown correspond to the 10 best models (i.e., with the lowest AICc) with their corresponding estimates (mean ± standard error). AICc differences (ΔAICc) between the best model (Model 1) and the rest of candidate models are also shown. In every model, individual was included as a random factor and environmental variables were standardized. Abbreviations as in Text S1. † *p*<0.1 ** *p*<0.001. Variable estimates without symbols were non-significant (*p*<0.1).

a) Home-range

| **Model** | **Intercept** | **Slope** | **%Shrub** | **%Rock** | **NF** | **ENN** | **AICc** | **ΔAICc** |
| --- | --- | --- | --- | --- | --- | --- | --- | --- |
| 1 | -0.079 ± 0.201 | -0.529 ± 0.138 ** | 0.666 ± 0.198 ** | 0.218 ± 0.136 | - | - | 457.4 | 0.000 |
| 2 | -0.043 ± 0.200 | -0.544 ± 0.137 ** | 0.518 ± 0.170 ** | - | - | - | 458.0 | 0.554 |
| 3 | -0.033 ± 0.202 | -0.606 ± 0.148 ** | 0.546 ± 0.178 ** | - | -0.153 ± 0.129 | - | 458.6 | 1.159 |
| 4 | -0.067 ± 0.203 | -0.567 ± 0.151 ** | 0.658 ± 0.201 ** | 0.183 ± 0.147 | -0.088 ± 0.139 | - | 459.1 | 1.653 |
| 5 | -0.069 ± 0.212 | -0.521 ± 0.146 ** | 0.665 ± 0.198 ** | 0.226 ± 0.148 | - | -0.021 ± 0.142 | 459.5 | 2.034 |
| 6 | -0.077 ± 0.210 | -0.565 ± 0.144 ** | 0.535 ± 0.175 ** | - | - | 0.064 ± 0.131 | 459.8 | 2.358 |
| 7 | 0.030 ± 0.232 | -0.603 ± 0.148 ** | 0.529 ± 0.181 ** | - | -0.231 ± 0.187 | -0.108 ± 0.190 | 460.3 | 2.892 |
| 8 | 0.021 ± 0.233 | -0.557 ± 0.151 ** | 0.648 ± 0.203 ** | 0.204 ± 0.149 | -0.194 ± 0.191 | -0.158 ± 0.195 | 460.5 | 3.065 |
| 9 | 0.110 ± 0.172 | -0.741 ± 0.123 ** | - | - | - | - | 466.9 | 9.488 |
| 10 | 0.118 ± 0.173 | -0.794 ± 0.135 ** | -0.125 ± 0.124 ** | - | - | - | 467.9 | 10.45 |

b) Within home-range

| **Model** | **Intercept** | **Slope** | **%Shrub** | **%Rock** | **NF** | **ENN** | **AICc** | **ΔAICc** |
| --- | --- | --- | --- | --- | --- | --- | --- | --- |
| 1 | -0.652 ± 0.394 † | - | 0.308 ± 0.174 † | - | - | - | 231.9 | 0.000 |
| 2 | -0.547 ± 0.370 | - | - | - | - | - | 233.0 | 1.115 |
| 3 | -0.441 ± 0.393 | - | - | - | - | -0.251 ± 0.183 | 233.1 | 1.181 |
| 4 | -0.563 ± 0.414 | - | 0.257 ± 0.182 | - | - | -0.172 ± 0.193 | 233.2 | 1.248 |
| 5 | -0.655 ± 0.391 † | - | 0.295 ± 0.175 † | - | 0.126 ± 0.169 | - | 233.4 | 1.510 |
| 6 | -0.668 ± 0.392 † | - | 0.371 ± 0.204 † | 0.126 ± 0.207 | - | - | 233.6 | 1.695 |
| 7 | -0.657 ± 0.404 | 0.011 ± 0.210 | 0.313 ± 0.203 | - | - | - | 234.0 | 2.061 |
| 8 | -0.555 ± 0.368 | - | - | - | 0.155 ± 0.165 | - | 234.2 | 2.284 |
| 9 | -0.504 ± 0.382 | -0.153 ± 0.179 | - | - | - | - | 234.3 | 2.414 |
| 10 | -0.561 ± 0.412 | - | 0.342 ± 0.205 † | 0.201 ± 0.220 | - | -0.227 ± 0.204 | 234.4 | 2.499 |
