# Supplementary material for: Effects of Frugivore Preferences and Habitat Heterogeneity on Seed Rain: A Multi-Scale Analysis
Source: PLoS One. 2012 Mar 16;7(3):e33246. doi: 10.1371/journal.pone.0033246 (PMC3306386; doi:10.1371/journal.pone.0033246)
Supplement: Table S2 — Effect of covariance structure on the spatial autocorrelation of model residuals. (DOC) [file pone.0033246.s010.doc]

**Table S2 – Effect of covariance structure on the spatial autocorrelation of model residuals**

Spatial autocorrelation of the residuals (Mantel-r and associated p-value, based on 10^3^ permutations computed with *vegan* library within the R environment) [1] and predictive power (AUC value) of Generalized Linear (Mixed) Models (GLMs) calculating lizard habitat preferences at two different scales: home-range and within home-range. Different models incorporate alternative covariance structures, aimed at capturing the residuals’ autocorrelation (see Fig S5) and therefore avoiding biases in parameter estimates and significance tests [2-5]. Because all these models assign spatial effects to an error term, they also retain autocorrelation in the residuals but, if adequate, they are tolerant of such autocorrelation and provide precise estimates and correct error rates; however, the use of an inappropriate covariance structure may also result in comparable or larger biases in parameter estimates and significance levels [6]. Models differed considerably in their parameter estimates and significance levels (data not shown); however, owing to their complex structure, we were not able to compare their fits based on AICc coefficients (GLMs that incorporate spatial covariance are fitted using penalized quasi-likelihood, e.g. in the *glmmPQL* library [7] within the R environment [8], and do not provide reliable AICc estimates). Hence, and because the primary purpose of our GLMs was to simulate the effect of lizard habitat preferences on seed dispersal, we decided to select the best model on the base of their predictive power, as estimated by the AUC (right column).

| **GLM** | **Spatial-correlation structure introduced in the model** | **Mantel-r** | ***p*-value** | | **AUC (± SD)** |
| --- | --- | --- | --- | --- | --- |
| Home range | No correlation | 0.105 | <0.001 | | 0.739±0.026 |
|  | Exponential | 0.087 | <0.001 | | 0.672±0.028 |
|  | Gaussian | 0.112 | <0.001 | | 0.697±0.028 |
|  | Lineal | 0.120 | <0.001 | | 0.708±0.027 |
|  | Ratio | 0.096 | <0.001 | | 0.689±0.028 |
|  | Spherical | 0.084 | <0.001 | | 0.628±0.029 |
| Within home-range | No correlation | +0.037 | | 0.104 | 0.749±0.037 |
|  | Exponential | -0.019 | | 0.403 | 0.558±0.044 |
|  | Gaussian | -0.018 | | 0.424 | 0.558±0.044 |
|  | Lineal | -0.017 | | 0.473 | 0.558±0.044 |
|  | Ratio | -0.019 | | 0.391 | 0.558±0.044 |
|  | Spherical | -0.017 | | 0.454 | 0.558±0.044 |

**References**

1. Oksanen J, Blanchet FG, Kindt R, Legendre P,, O'Hara RB, et al. (2010). vegan: Community Ecology Package. R Packaged version 1.17-4. <http://CRAN.R-project.org/package=vegan>
2. Legendre P, Dale, MRT, Fortin MJ, Gurevitch J, Hohn M, et al. (2002) The consequences of spatial structure for the design and analysis of ecological field surveys. Ecography 25: 601-615.
3. Fortin MJ, Dale MRT (2005) Spatial analysis: a guide for ecologists. Cambridge Univ Press, Cambridge, UK.
4. Dormann CF, McPherson JM, Araujo MB, Bivand R, Bolliger J, et al. (2007) Methods to account for spatial autocorrelation in the analysis of species distributional data: a review. Ecography 30: 609-628.
5. Peres-Neto PR Legendre P (2010) Estimating and controlling for spatial structure in the study of ecological communities. Global Ecol Biogeogr 19: 174-184.
6. Beale, CM Lenon, JL Yearsley JM, Brewer MJ, Elston DA (2010) Regression analysis of spatial data. Ecol Letters 13:246-264.
7. Broström G (2009). *glmmML*: Generalized linear models with clustering. R package version 0.81-6. <http://CRAN.R-project.org/package=glmmML>
8. R Development Core Team (2009) R: A language and environment for statistical computing. (ed) R Foundation for Statistical Computing, Vienna, Austria.
